# Non-Coding RNAs in Neurological and Neuropsychiatric Disorders: Unraveling the Hidden Players in Disease Pathogenesis

**DOI:** 10.3390/cells13121063

**Published:** 2024-06-19

**Authors:** Mirolyuba Simeonova Ilieva

**Affiliations:** The Bartholin Institute, Department of Pathology, Rigshospitalet, Copenhagen N, Ole Maaløes Vej 5, 3rd Floor, 2200 Copenhagen, Denmark; mirolyuba.simeonova.ilieva@regionh.dk or mirolyubailieva@gmail.com

**Keywords:** non-coding RNA, microRNA, long non-coding RNA, circular RNA, neurological disorders, neuropsychiatric disorders, Alzheimer’s disease, Parkinson’s disease, stroke, amyotrophic lateral sclerosis, multiple sclerosis, autism spectrum disorder, schizophrenia, depression, brain tumors, mechanisms, diagnosis, therapy

## Abstract

Neurological and neuropsychiatric disorders pose substantial challenges to public health, necessitating a comprehensive understanding of the molecular mechanisms underlying their pathogenesis. In recent years, the focus has shifted toward the intricate world of non-coding RNAs (ncRNAs), a class of RNA molecules that do not encode proteins but play pivotal roles in gene regulation and cellular processes. This review explores the emerging significance of ncRNAs in the context of neurological and neuropsychiatric disorders, shedding light on their diverse functions and regulatory mechanisms. The dysregulation of various ncRNAs, including microRNAs (miRNAs), long non-coding RNAs (lncRNAs), and circular RNAs (circRNAs), has been implicated in the pathophysiology of conditions such as Alzheimer’s disease, Parkinson’s disease, schizophrenia, and mood disorders. This review delves into the specific roles these ncRNAs play in modulating key cellular processes, including synaptic plasticity, neuroinflammation, and apoptosis, providing a nuanced understanding of their impact on disease progression. Furthermore, it discusses the potential diagnostic and therapeutic implications of targeting ncRNAs in neurological and neuropsychiatric disorders. The identification of specific ncRNA signatures holds promise for the development of novel biomarkers for early disease detection, while the manipulation of ncRNA expression offers innovative therapeutic avenues. Challenges and future directions in the field are also considered, highlighting the need for continued research to unravel the complexities of ncRNA-mediated regulatory networks in the context of neurological and neuropsychiatric disorders. This review aims to provide a comprehensive overview of the current state of knowledge and stimulate further exploration into the fascinating realm of ncRNAs in the brain’s intricate landscape.

## 1. Introduction

Brain disorders encompass a wide spectrum of conditions affecting the structure and function of the central nervous system (CNS). These disorders, including neurodevelopmental disorders (e.g., autism spectrum disorder), neurodegenerative diseases (e.g., Alzheimer’s disease), and neuropsychiatric disorders (e.g., schizophrenia), together with brain tumors, pose significant challenges to healthcare systems worldwide due to their complex etiology and limited treatment options. While protein-coding genes have traditionally been the focus of research in brain disorders, emerging evidence suggests that non-coding RNAs (ncRNAs) play crucial roles in their pathogenesis.

Non-coding RNAs, once considered transcriptional “noise”, are now recognized as key regulators of gene expression at the transcriptional, post-transcriptional, and epigenetic levels. MicroRNAs (miRNAs), long non-coding RNAs (lncRNAs), and circular RNAs (circRNAs) are among the most studied classes of ncRNAs implicated in brain disorders. Dysregulation of these ncRNAs has been associated with various aspects of CNS development, function, and pathology.

This article provides an overview of the current understanding of ncRNAs in brain disorders, focusing on their roles in disease pathogenesis, diagnosis, and therapeutic interventions. The recent advancements in ncRNA research are discussed, and the potential of these molecules as diagnostic biomarkers and therapeutic targets in the management of brain disorders are highlighted.

## 2. Non-Coding RNAs: Mechanisms of Action and Roles in Brain Molecular and Cellular Events

**Figure 1:**
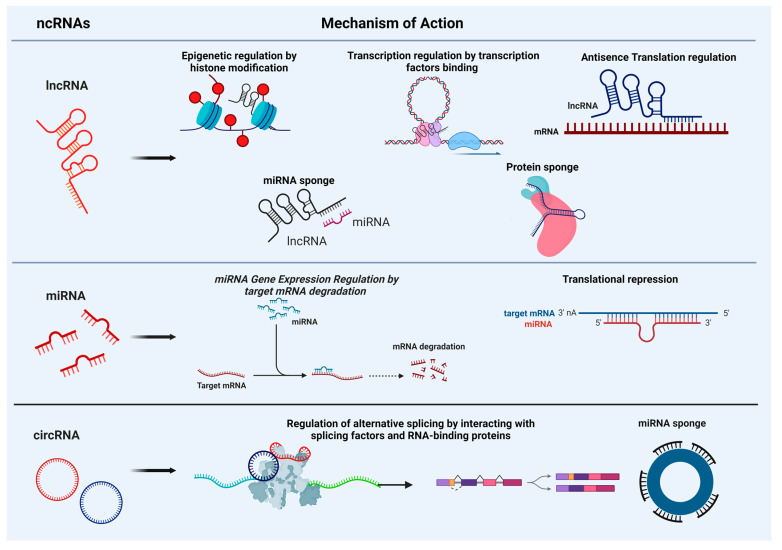
Schematic presentation illustrating the multifaceted mechanisms of action of long non-coding RNA (lncRNA), microRNA (miRNA), and circular RNA (circRNA). LncRNA can play a regulatory role by acting as epigenetic regulators, influencing chromatin remodeling and histone modification; scaffolds for the assembly of transcriptional regulatory complexes; regulating gene expression by forming RNA duplex with mRNA and modulating translation; miRNA and protein sponges. MiRNA functions as a post-transcriptional regulator by binding to target mRNA, leading to mRNA degradation or translational repression. CircRNAs regulate alternative splicing by interacting with splicing factors or RNA-binding proteins, acting as miRNA sponges, thus sequestering miRNAs and preventing them from targeting mRNA transcripts. (Figure generated by Biorender).

The capacity of non-coding RNAs (ncRNAs) to recognize and specifically interact with complementary sequences enables them to function as modulators of epigenetics through chromatin remodeling or to regulate gene expression at both the transcriptional and post-transcriptional levels. This ability triggers various cellular and molecular events related to normal and pathological processes in the brain (Figure 1).

*MicroRNAs (miRNAs)* are small, endogenous ncRNAs (~22 nucleotides) that regulate gene expression by binding to complementary sequences in the 3′ untranslated regions (UTRs) of target mRNAs [1]. By this binding, miRNAs can inhibit protein synthesis or promote mRNA decay, thereby regulating gene expression post-transcriptionally [2].

MiRNA genes may reside within intragenic regions, where they share transcriptional regulatory units with host genes, or they can be situated in intergenic regions of the genome, possessing their own independent cis-regulatory elements (CREs) [3].

The canonical pathway for miRNA production begins with the transcription of pri-miRNAs, which are then processed into pre-miRNAs by the microprocessor complex comprising an RNA-binding protein DiGeorge Syndrome Critical Region 8 (DGCR8) and a ribonuclease III enzyme, Drosha. DGCR8 recognizes specific motifs within the pri-miRNA, while Drosha cleaves the pri-miRNA duplex at its characteristic hairpin structure. After processing, pre-miRNAs are exported to the cytoplasm, where Dicer further processes them into mature miRNA duplexes. The directionality of the miRNA strand determines its name, with the 5p strand originating from the 5′ end of the pre-miRNA hairpin and the 3p strand from the 3′ end. Factors such as thermodynamic stability and sequence characteristics influence the selection of the 5p or 3p strand. The selected guide strand is loaded into Argonaute (AGO) proteins, while the passenger strand is unwound and degraded, resulting in the formation of mature miRNAs [1].

Mature miRNAs are distributed across various subcellular compartments in the cytoplasm, including RNA granules, endomembranes, and mitochondria, and they can be released from cells via exosomes. Emerging research has shown that mature miRNAs can additionally be found within the nucleus, suggesting a potential role in epigenetic regulation [4].

The mechanistic understanding of several miRNAs regarding their involvement in molecular and cellular events in the brain, as well as their potential connection to pathological processes, has been elucidated (Table 1).

**MiR-132** plays a critical role in orchestrating neuronal differentiation and maturation during neurodevelopment. It targets key transcription factors involved in neuronal fate determination and dendritic morphogenesis, such as MeCP2, p250GAP, CREB, and FOXO3, thereby promoting neurite outgrowth, dendritic arborization, and synapse formation [5,6,7,8].

Further, miR-132 targets synaptic proteins (e.g., RhoA) and ion channels (e.g., GABA receptors), regulating synaptic strength, neuronal connectivity, and circuit function [9,10,11].

In response to neuroinflammatory stimuli or pathological conditions, miR-132 participates in the regulation of glial activation and neuroinflammatory responses. It targets genes involved in microglial activation (e.g., EPAC1) and cytokine signaling (e.g., IL-6), modulating the production of pro-inflammatory cytokines and neurotoxic factors [12,13,14].

MiR-132 plays a key role in inter-tissue communication and inflammation regulation. The brain suppresses peripheral inflammation via vagal secretion of acetylcholine (ACh), and miR-132, which targets acetylcholinesterase (*AChE*), can attenuate inflammation. Inflammatory stimuli induce overexpression of miR-132 in leukocytes, and anti-miR-132 oligonucleotides reduce miR-132 levels while increasing AChE in mice. Experiments showed that miR-132 binding to AChE mRNA suppresses its expression, and transgenic mice with null AChE 3′UTR exhibited excessive inflammation and impaired cholinergic regulation despite miR-132 upregulation. These findings highlight miR-132 as a regulator of neuro-immune communication and a potential target for therapeutic intervention [15,16]. Additionally, MiR-132 targets genes involved in apoptosis (e.g., *p300*) and oxidative stress response (e.g., *FOXO3*), thus acting as a neuroprotective factor promoting neuronal survival, mitochondrial function, and antioxidant defense mechanisms [5].

**MiR-124** targets key transcription factors involved in neural stem cell maintenance (e.g., *Sox9*) and neuronal differentiation (e.g., *PTBP1*), thereby promoting the transition of neural progenitor cells into mature neurons [17,18].

By modulating the expression of genes involved in synaptic transmission, dendritic morphology, and neuronal excitability, miR-124 exerts profound effects on synaptic plasticity and neuronal function. It targets synaptic proteins (e.g., *LIMK1*, *NRXN1*) and ion channels (e.g., *KCC2*), influencing synaptic strength, neuronal connectivity, and circuit function [19,20].

MiR-124 participates in the regulation of glial activation and neuroinflammatory responses by targeting genes involved in microglial activation (e.g., *C/EBP-α*) and astrocyte reactivity (e.g., *GFAP*), modulating the production of pro-inflammatory cytokines and neurotoxic factors [21,22]. Further, miR-124 acts as a tumor suppressor in various brain tumors, including glioblastoma and medulloblastoma, by targeting oncogenic signaling pathways and promoting tumor cell differentiation and apoptosis. It inhibits the expression of genes involved in cell proliferation (e.g., *CDK6*), survival (e.g., *BCL2L12*), and invasion (e.g., *MMP-9*), thereby suppressing tumor growth and metastasis [23,24].

**MiR-137** plays a pivotal role in regulating neural stem cell (NSC) proliferation and differentiation during neurodevelopment and fine-tunes the balance between self-renewal and differentiation. It targets key transcription factors such as *Ezh2*, *Sox2*, and *Tbr2*, which are involved in maintaining NSC self-renewal and neuronal lineage commitment [25,26].

Beyond its role in neurodevelopment, miR-137 continues to exert influence on synaptic function and plasticity in the mature brain, contributing to cognitive function and behavior. It targets several components of synaptic signaling pathways, including NMDA receptors (*NRG1*), AMPA receptors (*GRIN2A*), and synaptic scaffolding proteins (*PSD95*), thereby regulating synaptic transmission and plasticity [27,28].

**MiR-155** serves as a master regulator of neuroinflammation, modulating the activation and function of glial cells, including microglia and astrocytes. Upon activation, microglia and astrocytes upregulate miR-155 expression, which in turn amplifies the inflammatory response by targeting negative regulators of immune activation, such as *SOCS1* and *SHIP1* [29,30].

Emerging evidence suggests that miR-155 plays a crucial role in maintaining blood–brain barrier (BBB) integrity and function, regulating endothelial cell activation and permeability in response to inflammatory stimuli. Upregulated miR-155 expression disrupts BBB integrity by targeting tight junction proteins (e.g., claudin-1, ZO-1), promoting endothelial cell dysfunction and increased vascular permeability [31,32]. This disruption of BBB integrity facilitates the infiltration of immune cells and circulating inflammatory factors into the brain parenchyma, exacerbating neuroinflammation and neuronal damage in brain disorders characterized by BBB dysfunction, such as cerebral ischemia, traumatic brain injury, and neuroinflammatory conditions.

**MiR-9** plays an essential role in orchestrating neurogenesis and neural differentiation during embryonic development and adult neurogenesis. It targets key transcription factors involved in neural stem cell maintenance (e.g., TLX, FOXG1) and neuronal differentiation (e.g., REST), thereby regulating the balance between progenitor cell proliferation and neuronal fate determination [33,34].

MiR-9 targets genes involved in microglial activation (e.g., NF-κB signaling pathway) and astrocyte reactivity (e.g., *GFAP*), modulating the production of pro-inflammatory cytokines and neurotoxic factors [35].

Further, miR-9 influences epigenetic regulation by affecting chromatin-modifying enzymes and transcriptional regulators implicated in gene expression control. It regulates the expression of histone deacetylases (e.g., *HDAC5*) and methyl-CpG binding proteins (e.g., *MECP2*), modulating chromatin remodeling and transcriptional activity [36].

**MiR-134** directs synaptic proteins such as LIMK1 and CREB, influencing synaptic strength, neuronal connectivity, and circuit function [37,38,39]. It targets key regulators of cytoskeletal dynamics (e.g., *LIMK1, Pumilio*), thereby modulating actin remodeling and dendritic arborization [40,41]. Additionally, miR-134 targets proteins involved in GABAergic signaling and glutamatergic neurotransmission, shaping the balance of excitatory and inhibitory neurotransmission [42,43].

*P-element-induced wimpy testis (PIWI)-interacting RNAs (piRNAs) *are 26–31 nt in length and are the most diverse class of small non-coding RNAs (sncRNAs). They interact with Piwi proteins to form RNA–protein complexes that target transposable elements (TEs) and regulate chromatin structure, DNA methylation, and histone modifications in neurons, thus playing a role in epigenetic regulation and transposon silencing in the brain [44,45,46,47]. Dysregulated piRNA-mediated transposon silencing may lead to genomic instability, DNA damage, and neuronal dysfunction, potentially contributing to the pathogenesis of neurodevelopmental disorders such as Rett syndrome and fragile X syndrome, which are associated with mutations in genes involved in DNA methylation and chromatin regulation [48,49,50].

Emerging evidence suggests that piRNAs play a role in regulating neuroinflammatory responses and immune signaling in the brain. They modulate the expression of genes involved in microglial activation, cytokine production, and inflammatory signaling pathways, influencing neuroinflammation and glial activation [51].

Furthermore, piRNAs regulate neuronal plasticity and synaptic function by controlling the expression of genes involved in dendritic morphogenesis, synaptic transmission, and synaptic plasticity. They interact with RNA-binding proteins and translational regulators, influencing mRNA stability, translation efficiency, and protein synthesis in neurons [52,53]. Dysregulated piRNA-mediated synaptic regulation may disrupt neuronal connectivity, circuit function, and synaptic homeostasis.

*Long Non-Coding RNAs (lncRNAs)* are a heterogeneous group of transcripts longer than 200 nucleotides that lack protein-coding potential. In mammals, lncRNAs can be transcribed from intergenic, exonic, or distal protein-coding regions of the genome by RNA polymerase II [54]. LncRNAs, often described as ‘mRNA-like’ due to splicing and polyadenylation, can also be non-polyadenylated or expressed from Pol I and Pol III promoters or processed from introns and repetitive elements. Based on their location relative to protein-coding genes, lncRNAs are classified as intergenic, intronic, sense, and antisense.

Natural antisense transcripts (NATs) are complementarily overlapping with other transcripts, whether protein-coding or non-coding. The majority of paired transcripts consist of non-coding to non-coding or non-coding to protein-coding pairs. Therefore, NATs are classified based on their genomic position relative to their paired transcripts, either in cis or in trans. Cis-NAT pairs originate from the opposite strand of the same genomic locus and exhibit perfect complementarity with the opposite strand transcript. In contrast, trans-NAT pairs arise from different genomic loci, and while the two RNA molecules may hybridize with each other, their sequence complementarity is imperfect [55].

Long intergenic non-coding RNA (LincRNAs) are non-coding RNA transcripts that make up most of the lncRNAs. Despite not coding for proteins, they have a structure similar to protein-coding genes, i.e., exon–intron–exon structure. Their functions are largely unknown, but they can regulate gene expression by influencing nuclear architecture and chromatin topology and acting as scaffolds for proteins and RNAs. LincRNAs also regulate neighboring gene transcription, encode functional micropeptides, and act as decoys [56]. LncRNAs also include circular RNAs from back-splicing and trans-acting regulatory RNAs from mRNA 3′ untranslated regions, all with various functional roles.

LncRNAs exhibit cell-specific expression patterns and are often localized within specific subcellular compartments, highlighting their role in defining cell identity and developmental trajectories. Their expression is dynamically regulated during differentiation across various cell types and tissues, reflecting their involvement in diverse biological processes, including chromatin remodeling, transcriptional regulation, and RNA processing [57]. LncRNAs can regulate gene expression by interacting with chromatin-modifying complexes, such as polycomb repressive complexes (PRCs) or histone acetyltransferases (HATs), to modulate chromatin structure and gene accessibility [58,59]. This interaction can result in either gene activation or repression, depending on the specific lncRNA and its genomic location.

LncRNAs can also act as scaffolds for the assembly of transcriptional regulatory complexes, bringing together transcription factors, co-activators, and chromatin modifiers to regulate target gene expression [60].

Additionally, lncRNAs can regulate transcriptional elongation or termination by influencing RNA polymerase II (RNAPII) activity or chromatin looping interactions [58,59,61]. In the CNS, lncRNAs regulate neuronal differentiation, synaptic plasticity, and neuroinflammation (Table 2).

BACE1-AS (ACE1-antisense transcript) is transcribed from the antisense strand of the β-site amyloid precursor protein-cleaving enzyme 1 (*BACE1*) gene, which encodes the β-secretase enzyme involved in the production of amyloid-β (Aβ) peptides implicated in AD pathogenesis. BACE1-AS regulates *BACE1* expression through a cis-acting mechanism, forming RNA duplexes with BACE1 mRNA and modulating its stability and translation [62]. Upregulated BACE1-AS expression may alter BACE1 levels and activity, leading to aberrant Aβ production and accumulation, a hallmark of Alzheimer’s disease pathology.

NEAT1 (Nuclear-Enriched Abundant Transcript 1) is a key architectural component of nuclear bodies known as paraspeckles, which play a crucial role in regulating gene expression and nuclear organization. Within paraspeckles, NEAT1 interacts with RNA-binding proteins (e.g., SFPQ) and transcription factors, modulating transcriptional activity, RNA processing, and mRNA splicing [63,64,65,66].

NEAT1 is implicated in the development and progression of brain tumors, particularly gliomas. It promotes glioma cell proliferation, invasion, and resistance to apoptosis by modulating cell cycle progression, epithelial–mesenchymal transition (EMT), and DNA damage response pathways [67]. Overexpression of NEAT1 in gliomas correlates with tumor grade, progression, and patient prognosis, highlighting its potential as a therapeutic target for glioma treatment.

Long intergenic non-coding RNA HOTAIR (HOX transcript antisense RNA) functions as a scaffold for chromatin-modifying complexes, such as polycomb repressive complex 2 (PRC2) and lysine-specific demethylase 1 (LSD1), facilitating their recruitment to specific genomic loci [68]. Through these interactions, HOTAIR modulates histone methylation and acetylation patterns, leading to changes in chromatin structure and gene expression. Dysregulation of HOTAIR-mediated epigenetic regulation may alter the transcriptional landscape in the brain, contributing to the pathogenesis of neurodevelopmental disorders and neurodegenerative diseases.

Additionally, HOTAIR is involved in the regulation of neuroinflammatory responses and glial activation in the brain and could be a potential therapeutic target for multiple sclerosis and stroke [69,70].

*Circular RNAs (circRNAs)* are covalently closed, single-stranded RNA molecules formed by back-splicing of pre-mRNA transcripts. CircRNA expression is stable because it is not easily degraded by RNA exonucleases. CiRNAs have a wide range of origins and tissue specificities. There are five main origins of circRNAs: exon-only circRNA; intron-only circRNA; back-splicing of upstream exons and intron retention; circRNA from Fusion Gene; and read-through circRNA formed by polymerase II [71]. CircRNAs can act as microRNA sponges, sequestering miRNAs away from their target mRNAs and thereby relieving the inhibitory effect of miRNAs on gene expression [72]. This interaction between circRNAs and miRNAs can regulate the abundance and activity of miRNAs in the cell.

Additionally, circRNAs can regulate alternative splicing of pre-mRNA transcripts by interacting with splicing factors or RNA-binding proteins, leading to the generation of different mRNA isoforms with distinct functions [73]. CircRNAs are enriched in the brain and exhibit cell type-specific and developmental stage-specific expression patterns. Emerging evidence suggests that circRNAs play regulatory roles in synaptic plasticity, neuronal development, and neurodegeneration [74,75]. Dysregulated circRNA expression has been implicated in brain disorders such as Huntington’s disease (HD) and amyotrophic lateral sclerosis (ALS).

CiRS-7 (Cdr1as) acts as a sponge for miR-7, a microRNA involved in regulating neuronal development and synaptic function. By sequestering miR-7, Cdr1as indirectly modulates the expression of miR-7 target genes, influencing processes such as neuronal survival and inflammation [76,77].

CircHIPK3 has been implicated in neuronal differentiation and synaptic plasticity. It regulates the expression of genes involved in neuronal development and function, potentially contributing to brain development. High circHIPK3 expression is induced after ischemic stroke, and knockdown of circHIP3K alleviates cerebral ischemia–reperfusion (I/R) injury [78]. CircHIPK3 functions as an endogenous sponge of miR-148b-3p to upregulate *CDK5R1*. MiR-148b-3p alleviates cerebral I/R injury targets via CDK5R1/SIRT1 axis. Thus, circHIPK3 exacerbates cerebral I/R injury via miR-148b-3p/CDK5R1/SIRT1 axis [78].

CircHIPK3 overexpression alleviated oxygen–glucose deprivation (OGD)-induced AGE1.HN cell (derived from neural precursor tissue) inflammatory response and neuronal apoptosis via regulating miR-382-5p/DUSP1 axis, indicating that circHIPK3 might be a promising therapeutic target for spinal cord injury [79]. CircHIPK2 functions as an endogenous microRNA-124 (MIR124–2HG) sponge to sequester MIR124–2HG and inhibit its activity, resulting in increased sigma non-opioid intracellular receptor 1 (SIGMAR1/OPRS1) expression [80]. Knockdown of circHIPK2 expression significantly inhibited astrocyte activation via the regulation of autophagy and endoplasmic reticulum (ER) stress through the targeting of MIR124–2HG and SIGMAR1 [80]. These findings were confirmed in vivo in mouse models, as microinjection of a circHIPK2 siRNA lentivirus into mouse hippocampi inhibits astrocyte activation induced by methamphetamine or lipopolysaccharide (LPS). These findings provide novel insights regarding the specific contribution of circHIPK2 to astrocyte activation in the context of drug abuse as well as for the treatment of a broad range of neuroinflammatory disorders.

CircDLGAP4 acts as a natural sponge for microRNA-143 (miR-143), suppressing miR-143 activity and thereby inhibiting the expression of Homologous to the E6-AP C-Terminus (HECT) E3 Ubiquitin Protein Ligase 1. Reduced levels of circDLGAP4 are observed in the plasma of acute ischemic stroke patients. Elevating circDLGAP4 expression significantly mitigates neurological deficits, reduces infarct size, and mitigates blood–brain barrier damage in a mouse model of transient middle cerebral artery occlusion-induced stroke. Furthermore, endothelial–mesenchymal transition contributes to blood–brain barrier disruption, and circDLGAP4 overexpression effectively inhibits this transition by regulating the expression of tight junction proteins and mesenchymal cell markers [81].

CircRNA_FMR1 is derived from the FMR1 gene, mutations of which are associated with fragile X syndrome. CircRNA_FMR1 has been implicated in the regulation of synaptic plasticity and cognitive function, suggesting a potential role in the pathogenesis of fragile X syndrome [82].

NcRNAs can undergo various post-transcriptional modifications, such as RNA editing, methylation, or adenosine deamination, which can affect their stability, localization, and activity. For example, adenosine-to-inosine (A-to-I) RNA editing can alter the sequence and structure of ncRNAs, leading to changes in their binding affinity or target specificity [83].

These RNA modifications can modulate the function of ncRNAs in gene regulation and cellular processes, providing an additional layer of complexity to the regulatory networks in the brain.

**Figure 2:**
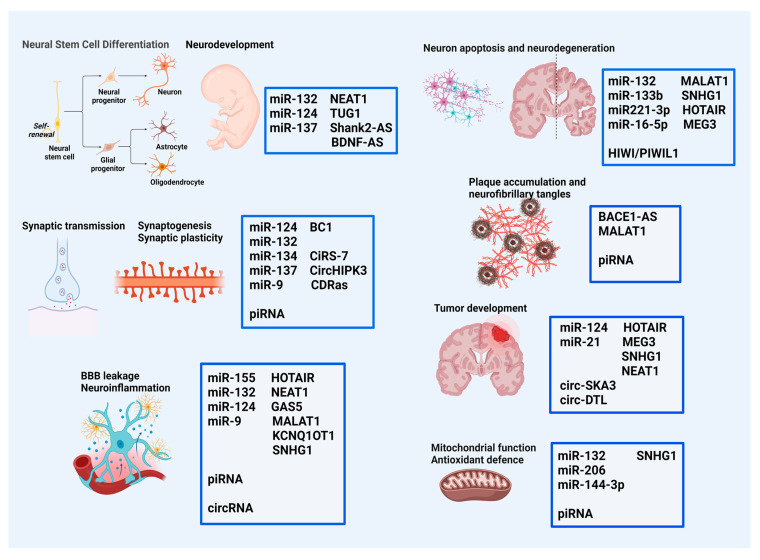
Role of ncRNAs in brain molecular and cellular events and pathology. NcRNAs orchestrate diverse molecular and cellular processes in the brain, including neural stem cell differentiation, synaptic transmission, neuroinflammation, blood–brain barrier integrity, apoptosis, neurodegeneration, plaque accumulation, tumor development, mitochondrial function, and antioxidant defense. Dysregulation of ncRNAs contributes to neurological disorders such as Alzheimer’s, Parkinson’s, and Huntington’s diseases, glioblastoma, and ischemic stroke, as well as neurodevelopmental disorders. (Figure generated by Biorender).

Overall, the mechanisms of action of ncRNAs in the brain are highly diverse and interconnected, involving multiple levels of regulation and interaction with various molecular factors. By modulating gene expression and cellular processes, ncRNAs contribute to brain development, synaptic plasticity, and the pathogenesis of neurological and neuropsychiatric disorders (Figure 2).

## 3. Role of ncRNAs in Specific Brain Disorders

### 3.1. Alzheimer’s Disease (AD)

Alzheimer’s disease (AD) is a progressive neurodegenerative disorder characterized by a gradual decline in cognitive function, memory loss, and behavioral changes. It is the most common cause of dementia, affecting primarily older adults, although early-onset forms can occur. AD is characterized by the accumulation of abnormal protein deposits in the brain, including beta-amyloid plaques and tau tangles, which lead to the death of nerve cells and subsequent brain atrophy. Dysregulation of ncRNAs has been implicated in the pathogenesis of AD, contributing to amyloid-β (Aβ) deposition, tau hyperphosphorylation, neuroinflammation, and synaptic dysfunction [14,84,85].

MiR-34a expression is increased in the temporal cortex of AD patients and correlates with the severity of AD pathology compared to age-matched healthy controls. This overexpression is linked to the repression of target genes involved in synaptic plasticity, oxidative phosphorylation, and glycolysis, resulting in reduced ATP production and glycolytic capacity. Analysis of the miR-34a gene promoter region reveals response elements for NFκB, STAT1, c-Fos, CREB, and p53, suggesting that activation of these transcription factors may drive miR-34a expression. This leads to the repression of critical genes, resulting in dysfunctions in synaptic plasticity, energy metabolism, and network activity [84].

The target genes of miR-124 and miR-132 are involved in Aβ production, tau protein phosphorylation, and microglia activation, which are key hallmarks of AD pathology [6,86].

The lncRNA BACE1-AS is upregulated in the brains of people with AD and might be detected in the bloodstream, making it a potential biomarker for the disease stage. Aβ peptides are produced from the proteolytic cleavage of APP by BACE1. *BACE1* transcripts are regulated by BACE1-AS, which is transcribed from the opposite strand of the *BACE1* gene and forms RNA duplexes with BACE1 mRNA. This interaction increases the stability and translation of BACE1 mRNA, enhancing BACE1 production in a positive feedback loop. Upregulation of BACE1-AS is crucial for increasing BACE1 half-life and promoting the production of disease-associated Aβ peptides [62].

Another lncRNA, MALAT1, is found to be protective against Aβ1–42-induced toxicity and is decreased in the cerebrospinal fluid (CSF) of AD patients. This protective effect may be due to MALAT1 sponging several miRNAs, incl. miR-200a, miR-26a, and miR-26b, which are naturally elevated in AD. These miRNAs target the *receptor tyrosine kinase (RTK) EPHA2* and several of its downstream effectors, which are protective against Aβ1–42-induced cytotoxicity [87].

In AD, increased expression of HIWI/PIWIL1 in hippocampal neurons is observed, likely due to global heterochromatin relaxation induced by pathogenic tau aggregates. This alteration correlates with the upregulation of numerous piRNAs in AD brains, many of which target complementary gene transcripts. Specifically, it has been demonstrated that 103 out of 9453 human brain piRNAs were differentially expressed in AD brains, with several correlating with genome-wide significant AD risk single nucleotide polymorphisms (SNPs) [88]. Moreover, 10 piRNAs have been identified to be significantly associated with years of survival, suggesting a potential role in lifespan determination [89].

### 3.2. Parkinson’s Disease (PD)

Parkinson’s disease (PD) is a chronic and progressive neurodegenerative disorder characterized primarily by motor symptoms such as tremors, rigidity, bradykinesia, and postural instability. It results from the loss of dopamine-producing neurons in the substantia nigra, a region of the brain that controls movement. Non-motor symptoms, including cognitive impairment, mood disorders, and sleep disturbances, are also common. PD is characterized by α-synuclein proteins misfolding, forming insoluble fibrils and intracellular inclusions known as Lewy bodies. NcRNAs influence α-synuclein aggregation, mitochondrial dysfunction, oxidative stress, and dopaminergic neuron degeneration, thus playing a crucial role in PD pathology. For example, miR-133b and miR221-3p have been found to be upregulated in the plasma of PD patients, affecting dopaminergic neuron survival and function, and could serve as an early noninvasive biomarker for PD diagnosis [90].

In PD, differentially expressed piRNAs in prefrontal cortex and amygdala tissues of PD patients have been identified, incl. piR-has-92056, piR-hsa-150797, piR-hsa-347751, piR-hsa-1909905, piR-hsa-2476630, and piR-hsa-2834636 [91]. These piRNAs were predicted to target protein-coding genes implicated in PD pathogenesis, such as Mitochondrially encoded *cytochrome C oxidase I* (*MT-CO1*) and *MT-CO3*. Moreover, six piRNAs identified in blood small extracellular vesicles showed promise as noninvasive biomarkers for PD diagnosis. However, how these modest levels of piRNAs affect their cognate genes is not clear and remains to be further verified.

lncRNAs, such as SNHG1, HOTAIR, and MALAT1, have been found to regulate dopaminergic neuron survival and inflammation in PD. SNHG1 has been found to be upregulated in Parkinson’s disease (PD), where it acts as a sponge for several miRNAs, including miR-7 [92], miR-125b-5p [93], miR-181a-5p [94], and miR-221/222/p27 [95]. This interaction affects downstream targets and signaling pathways such as NLRP3, MAPK1, CXCL12, and mTOR. Consequently, SNHG1 modulates gene expression associated with oxidative stress, apoptosis, autophagy, and neuroinflammation pathways, all of which are implicated in PD pathogenesis.

The lncRNA HOTAIR was found to be upregulated in the mouse midbrain, potentially promoting PD induced by MPTP. Overexpression of HOTAIR led to an increase in leucine-rich repeat kinase 2 (LRRK2) expression while knocking down HOTAIR decreased LRRK2 levels. HOTAIR knockdown offered protection against MPP+-induced DA neuronal apoptosis by inhibiting caspase 3 activity. Moreover, HOTAIR knockdown demonstrated a protective effect on the cell viability of SH-SY5Y cells treated with MPP+, which was abrogated by overexpression of LRRK2 [96,97]. Unlike in AD, where MALAT1 is downregulated, it was discovered that MALAT1 is upregulated in midbrain tissue of neurotoxin MPTP-induced PD mice, suggesting a potential role in PD pathogenesis. It was also observed that Malat1 binds to α-Synuclein, boosting its stability and resulting in elevated protein expression of α-Synuclein. Furthermore, β-asarone was found to protect neurons from MPTP-induced injury by reducing α-Synuclein expression. Interestingly, Malat1 overexpression reversed this protective effect [98].

### 3.3. Amyotrophic Lateral Sclerosis (ALS)

Amyotrophic Lateral Sclerosis (ALS) is a fatal motor neuron disease that progressively impairs neuronal cells responsible for controlling voluntary muscle activity. By targeting genes involved in motor neuron function, protein homeostasis, and mitochondrial function, ncRNAs contribute to ALS pathogenesis.

MiR-206 is highly expressed in skeletal muscles and is involved in ALS pathogenesis, regulating mRNA translation, protein aggregation, and oxidative stress [99]. In a mouse model of ALS expressing mutated human superoxide dismutase 1 (SOD1-G93A), miR-206 has been identified as a potential biomarker. MiRNA alterations were studied in the skeletal muscle and plasma of these mice, and miR-206 was found to be consistently altered during disease progression. Increased levels of miR-206 were observed in fast-twitch muscles of symptomatic SOD1-G93A mice, with the highest expression in the most severely affected animals. Additionally, miR-206 was elevated in the circulation of both symptomatic mice and human ALS patients. These findings suggest that miR-206 is a promising candidate biomarker for ALS, though larger-scale studies on human patients are needed to confirm its potential [100].

A key pathological feature of ALS is the aggregation of the TAR DNA-binding protein 43 (TDP-43) in neuronal cells, which suggests dysregulated RNA metabolism. In sporadic ALS brains, several piRNAs are differentially expressed: hsa-piR-000578, hsa-piR-020871, and hsa-piR-022184 are upregulated, while hsa-piR-009294 and hsa-piR-016735 are downregulated. HIWI/PIWIL1 expression is increased, and HIWI2/PIWIL4 is decreased in ALS brain tissues, with HIWI/PIWIL1 co-localizing with TDP-43 in motor neurons, possibly contributing to TDP-43 inclusions. Additionally, the piRNA hsa-piR-33151 is decreased in the serum of ALS patients. These findings suggest that piRNA dysregulation is linked to ALS pathogenesis and may serve as potential diagnostic biomarkers and therapeutic targets [101].

The complexity of ALS is partly due to various cellular features, including paraspeckles, which are nuclear bodies formed by specialized proteins and RNAs like the lncRNA NEAT1_2. NEAT1_2 is predominantly expressed in spinal motor neurons during the early stages of ALS and interacts with ALS-associated RNA-binding proteins such as TDP-43 and FUS/TLS. These proteins are enriched in paraspeckles and bind directly to NEAT1_2, as verified by iCLIP data. Both TDP-43 and FUS/TLS are essential for normal paraspeckle formation. Increased paraspeckle formation during the early phase of ALS suggests that NEAT1_2 may serve as a scaffold for RNA-binding proteins in ALS motor neurons. Additionally, FUS is integral to paraspeckle stability, regulating NEAT1 levels and maintaining the structure, with FUS mutations potentially impairing stress response mechanisms [102].

### 3.4. Huntington’s Disease (HD)

Huntington’s disease (HD), a genetic disorder, results in the gradual degeneration of brain nerve cells, resulting in motor control difficulties, cognitive decline, and psychiatric symptoms. It stems from a mutation in the *huntingtin (HTT)* gene, where an excess of CAG repeats (more than 36) triggers the formation of an unstable protein. These expanded repeats cause the production of a huntingtin protein with an unusually long polyglutamine tract at the N-terminus.

A small non-coding RNA sequencing (sncRNA-seq) data analysis from HD cortical prefrontal cortex tissues identified sixteen piRNAs with differential expression in HD brains. Target prediction and pathway enrichment analysis revealed potential target genes involved in brain pathophysiology, including *Caspase-8* (*CASP8*) and *Fas-associated protein with death domain* (*FADD*), *Neural precursor cell expressed developmentally downregulated gene 4-like (NEDD4L)*, and *SMAD2/3:SMAD4* [103]. Additionally, thirty-six miRNAs have been identified and associated with at least one of the KEGG pathways relevant to HD, including apoptosis, cellular senescence, ubiquitin-mediated proteolysis, and signaling pathways such as neurotrophin, p53, ErbB, Notch, MAPK, AMPK, PI3K-Akt, mTOR, Wnt, and Hippo. In addition, several miRNAs identified are known to interact directly with the *HTT* gene, including miR-16-5p and miR-107 [104,105]. Notably, miR-16-5p has been found to also directly interact with brain-derived neurotrophic factor (BDNF), a neurotrophin regulated by HTT. The dysregulation of BDNF expression, stemming from the altered structure of mutant HTT protein, may contribute to the progressive neuronal cell death observed in HD pathogenesis.

Moreover, miRNAs such as miR-144-3p have been implicated in metabolic regulation and mitochondrial function, with their aberrant expression potentially serving as a compensatory response to elevated levels of reactive oxygen species (ROS) in HD. Given that mitochondrial dysfunction is an early hallmark of HD pathogenesis and may contribute to neuronal dysfunction, miR-144-3p’s involvement underscores its relevance as a putative therapeutic target [106].

LncRNA NEAT1L, which can be inhibited by mutant huntingtin (mHTT) and MeCP2 via RNA–protein interactions, may play a protective role in CAG-repeat expansion. Twelve dysregulated lncRNAs were identified in R6/2 HD mice brains, eight of which have human homologs [107]. Among these, Meg3, Neat1, and Xist showed significant overexpression in cell and animal models of HD. Silencing Meg3 and Neat1 reduced mHTT aggregate formation and decreased Tp53 levels. Additionally, the lncRNA Abhd11os, which is downregulated in various HD animal models, has a neuroprotective effect when upregulated and a toxic effect when silenced, contributing to striatal vulnerability in HD [108].

A microarray-based study revealed an upregulation of the lncRNA NEAT1 in HD [109], and the finding was validated in R6/2 HD mouse models and postmortem human HD brain tissues. Functional studies demonstrated that NEAT1 increased cell viability under oxidative stress, suggesting its upregulation may be a neuroprotective response rather than a pathological event. Further research indicated that the long isoform of NEAT1 is also upregulated in HD, dependent on mutant huntingtin (mHTT). This isoform’s upregulation protects against mHTT-induced cytotoxicity, while its downregulation impairs cell proliferation and development. Additionally, dysregulated genes in HD overlap with pathways affected by NEAT1 downregulation, highlighting its potential role in HD pathology [110].

### 3.5. Multiple Sclerosis (MS)

Multiple sclerosis (MS) is a chronic autoimmune disease of the CNS characterized by inflammation, demyelination, and neurodegeneration. It results in a wide range of neurological symptoms, including fatigue, muscle weakness, impaired coordination, and cognitive dysfunction. The progression and severity of MS can vary greatly among individuals, and it may lead to significant disability over time.

NcRNAs play a crucial role by modulating immune responses, neuroinflammation, and myelin repair processes in MS.

MiR-155 was detected to be significantly upregulated in a cohort of MS patients. Increased expression correlates with increased IL-17, IFNγ, TNF, and IL-6, suggesting that miR-155 elevation occurs when cells are in an inflammatory state [111]. Mechanistically, miR-155 promotes pro-inflammatory responses in macrophages by inhibiting multiple targets (*SHIP1, FADD, SOCS1, IKK, IL13R1, CEBPβ*, and *SMAD2*), leading to increased pro-inflammatory cytokines and reactive oxygen species [112]. The interleukin 13 (IL-13) pathway in human macrophages is modulated by microRNA-155 via direct targeting of *interleukin 13 receptor alpha1* (*IL13Ralpha1*). Notably, miR-155 affects around 650 genes essential for M1 macrophage polarization, as demonstrated by a whole genome transcriptome study [113].

Additionally, miR-326 has been implicated in the regulation of neuroinflammation and demyelination in MS [114]. It modulates immune cell activation and differentiation and was upregulated in the blood samples of MS patients.

LncRNAs NEAT1 and KCNQ1OT1 are upregulated in MS patients, and this upregulation correlates with the disease’s inflammatory and neurodegenerative processes. NEAT1 acts as a positive regulator of inflammation, increasing the expression of pro-inflammatory cytokines and chemokines such as IL-6, CXCL10, TNF-α, and IL-17. KCNQ1OT1 binds to repressive complexes like polycomb repressive complex 2 (PRC2) and methyltransferase G9a, modulating gene expression. This epigenetic regulation affects immune cell function and inflammatory responses, which are crucial in MS pathogenesis. MS is characterized by an imbalance between pro-inflammatory Th17 cells and regulatory T cells (Tregs). The upregulation of NEAT1 and KCNQ1OT1 has been linked to a Th17/Treg imbalance by promoting Th17 cell differentiation and activity while suppressing Treg function, as well as increasing Th1-associated TNF-α and Th17-associated IL-17 activity. Interestingly, findings indicate that NEAT1 expression is higher in female MS patients compared to males, suggesting a sex-specific influence on immune system function and disease severity. This could help explain the higher incidence and severity of MS observed in women [115].

Additionally, lncRNAs HOTAIR and GAS5 have been detected upregulated in patients with MS, influencing T cell activation, microglial polarization, and oligodendrocyte differentiation. GAS5 is also considered a marker of MS severity [69,116].

### 3.6. Stroke

A stroke, medically known as a cerebrovascular accident (CVA), is a sudden interruption of blood supply to the brain, resulting in the loss of brain function. This interruption can occur due to a blockage of blood flow (ischemic stroke) or the rupture of a blood vessel (hemorrhagic stroke). Inflammation and cell death are pivotal components of the pathophysiological process. Immune cells like microglia and leukocytes become activated, releasing pro-inflammatory molecules and exacerbating the inflammatory response. Additionally, disruption of the blood–brain barrier allows infiltration of immune cells and inflammatory mediators into the brain parenchyma. Activated glial cells, namely astrocytes and microglia, contribute further by releasing reactive oxygen species and inflammatory molecules.

On the cellular level, ischemic strokes induce an energy failure in neurons due to oxygen and glucose deprivation, setting off cascades of events, including excitotoxicity, calcium overload, and oxidative stress, ultimately leading to neuronal death via apoptosis or necrosis. Excitotoxicity, driven by excessive glutamate release and oxidative stress from reactive oxygen and nitrogen species, plays a significant role in neuronal damage. Both apoptotic and necrotic pathways contribute to cell death.

A study found that increased expression of miR-155 in acute ischemic stroke patients was linked to elevated levels of JAK2/STAT3 and TNF-α, important inflammatory markers. miR-155 also correlated positively with ESR, an independent marker for poor stroke outcomes. The relationship between miR-155 and STAT3 was highlighted, suggesting a direct role of miR-155 in promoting post-stroke inflammation through the STAT3/JAK2 axis. The inflammatory response post-stroke, characterized by elevated inflammatory mediators and reduced neuroprotective factors, is associated with poor prognosis [117].

Studies consistently show that reduced MALAT1 expression increases cell death in endothelial cells after ischemia in vitro and worsens brain damage in mouse ischemic stroke (IS) models. A significantly decreased MALAT1 expression in IS patients was found, suggesting its potential as a prognostic indicator [118]. They also identified genetic variants associated with IS susceptibility, including the rs619586 polymorphism and rs1194338 SNP in MALAT1. While the rs619586 A allele correlates with lower MALAT1 expression, the rs1194338 AC/AA genotypes may act protectively against IS. Furthermore, MALAT1 expression negatively correlates with IS severity and pro-inflammatory factors, while it positively correlates with anti-inflammatory factors. Though the association with recurrence-free survival is not significant, there is a trend toward longer survival in patients with high MALAT1 expression. These findings highlight MALAT1’s potential as a prognostic marker and its involvement in IS pathogenesis and severity [119].

In a study of 181 cerebrovascular disease (CVS) patients, including chronic hypertensive and non-hypertensive stroke patients, lncNEAT1 levels were significantly elevated in both groups, especially in hypertensive patients, while lncHOTAIR levels were notably decreased in all cases, particularly in hypertensive stroke patients. Additionally, lncGAS5 levels were significantly reduced in both patient groups compared to controls, with a more pronounced decrease in hypertensive patients. Furthermore, NEAT1 and GAS5 levels were inversely correlated with stroke severity, while HOTAIR showed a positive correlation. The study suggests NEAT1, HOTAIR, and GAS5 could serve as diagnostic and prognostic biomarkers for CVS, particularly in hypertensive patients, and may represent potential therapeutic targets [120].

CircRNAs act as competing endogenous RNAs (ceRNAs) to sequester miRNAs involved in stroke-related pathways. For example, a binding site between circHIPK2 and miR-124 was discovered [80]. MiR-124 is involved in the regulation of stem cell differentiation and its ability to enhance the production of differentiated neurons from neural stem cells (NSC) [121]. Furthermore, research has shown that miR-124 exhibits a neuroprotective function and contributes to nerve recovery following stroke. Thus, silencing circHIPK2 in NSC (si-circHIPK2-NSCs) improved functional recovery and brain plasticity after stroke, indicating promising treatment potential. Additionally, the study revealed Smox as a downstream mediator of circHIPK2, regulating TUJ1 levels in differentiated neurons of NSCs. Smox downregulation significantly improved brain injury, suggesting its involvement in NSC differentiation. This study provides valuable insights into the therapeutic potential of si-circHIPK2-NSCs and the role of Smox in ischemic stroke, highlighting promising avenues for future research and treatment development [122].

### 3.7. Epilepsy

Epilepsy is a neurological disorder characterized by recurrent seizures, which are brief episodes of involuntary movement that may involve a loss of consciousness, convulsions, or abnormal sensory experiences. These seizures result from abnormal electrical activity in the brain and can vary widely in severity and frequency. Epilepsy can have various causes, including genetics, brain injury, infection, or developmental disorders. In epilepsy, dysregulation of specific miRNAs has been implicated in aberrant neuronal excitability and synaptic plasticity. For instance, MiR-134, a dendritically localized miRNA, controls synaptic Limk1 protein expression, influencing dendritic spine size by regulating Limk1 mRNA. In the absence of synaptic activity, miR-134 recruits a silencing complex, repressing Limk1 mRNA translation and limiting spine growth. Upon synaptic stimulation, BDNF activates the TrkB/mTOR pathway, inactivating the silencing complex, enhancing Limk1 synthesis, and promoting spine growth. Multiple miRNAs may collectively regulate Limk1 mRNA translation, with miR-134 targeting other neuronal mRNAs involved in synaptic development [39]. Upregulation of miR-134 has been observed in experimental models of epilepsy, suggesting its involvement in seizure generation and epileptogenesis [123].

In epilepsy, certain lncRNAs have emerged as key regulators of gene expression networks implicated in seizure susceptibility and neuronal dysfunction. For example, synaptodendritic BC1 RNA acts as a translational repressor in group I mGluR-stimulated pathways, regulating neuronal excitability. Downregulation of BC1 leads to neuronal hyperexcitability, increased cortical gamma-frequency oscillations, and heightened seizure susceptibility. These effects are reversed by protein synthesis inhibition, group I mGluR blockade, or ERK-MEK signaling inhibition. While the exact localization of the BC1 function is not determined, its presence in postsynaptic microdomains suggests a synaptodendritic role [124].

Dravet syndrome (DS), characterized by severe epilepsy, is often caused by mutations in the *SCN1A* gene. This study explored the expression of long non-coding RNAs (lncRNAs) related to SCN1A in brain tissues of pediatric epilepsy patients, aiming to identify potential targets for therapy. Two SCN1A-related lncRNAs, SCN1A-dsAS and SCN1A-usAS, were widely expressed in the brains of pediatric patients. SCN1A-dsAS showed a negative correlation, while SCN1A-usAS showed a positive correlation with SCN1A mRNA expression. Transfection experiments suggested that SCN1A-dsAS suppresses SCN1A mRNA generation. Targeting SCN1A-dsAS with antisense oligonucleotides (ASOs) could be a promising precision medicine approach to enhance SCN1A expression in DS therapy [125].

While the functional significance of circRNAs in epilepsy remains largely unexplored, emerging evidence suggests their involvement in modulating neuronal excitability and synaptic plasticity. For instance, circRNA CDR1as has been identified as a sponge for miR-7, a key regulator of synaptic transmission and neuronal survival. Knocking down of CDR1 in animal studies may contribute to aberrant synaptic function and seizure generation in epilepsy [126].

### 3.8. Brain Tumors

In glioblastoma, the most aggressive form of brain cancer, miRNAs stand as sentinel regulators of oncogenic pathways, modulating a plethora of target genes implicated in tumor proliferation, invasion, and angiogenesis. MiR-21, a prototypical oncogenic miRNA, reigns supreme in glioblastoma, promoting tumor growth and therapy resistance by targeting tumor suppressors such as *PTEN* and *PDCD4* [127]. Conversely, miR-124, a master regulator of neuronal differentiation, is often silenced in glioblastoma, unleashing a cascade of pro-tumorigenic events by derepressing oncogenic targets like *CDK6* and *SNAI2* [128,129]. The delicate balance between these opposing forces underscores the pivotal role of miRNAs in sculpting the malignant landscape of glioblastoma, offering exciting opportunities for therapeutic intervention.

Meanwhile, lncRNAs emerge as architects of epigenetic remodeling, dictating the chromatin landscape and transcriptional programs that govern glioma genesis. LncRNA HOTAIR, a potent regulator of chromatin dynamics, orchestrates a complex network of gene expression changes in glioblastoma, promoting tumor progression and therapy resistance through epigenetic silencing of tumor suppressors and activation of oncogenic pathways [130]. Conversely, lncRNA MEG3 exerts tumor-suppressive effects by inhibiting cell proliferation and inducing apoptosis in glioblastoma cells, offering a glimmer of hope amidst the darkness of malignant transformation [131].

Studies on various malignant brain tumor tissues have revealed elevated expression levels of *small nucleolar RNA host gene 1* (*SNHG1*), which correlates with the malignant progression and unfavorable prognosis of glioma. The underlying molecular mechanism involves *SNHG1* regulating the malignant behavior of glioma cells by interacting with microRNA-154-5p or miR-376b-3p. Additionally, *FOXP2* serves as a direct downstream target of both microRNA-154-5p and miR-376b-3p, leading to increased promoter activities and enhanced expression of the oncogenic gene *KDM5B*. Notably, KDM5B also functions as an RNA-binding protein to maintain the stability of SNHG1. This study highlights the significance of the SNHG1-microRNA-154-5p/miR-376b-3p-FOXP2-KDM5B feedback loop in regulating the malignant behavior of glioma cells [132].

LncRNA NEAT1 contributes to cancer growth through its effects on cell proliferation, migration, invasion, and drug resistance. NEAT1 functions as a competing endogenous RNA by binding to miR-324-5p, preventing its interaction with target mRNAs. One of these targets, *potassium channel tetramerization protein domain containing 20 (KCTD20*), is specifically regulated by miR-324-5p. Inhibition of NEAT1 leads to decreased levels of KCTD20 through competitive binding with miR-324-5p, resulting in reduced cell proliferation and increased apoptosis. Co-inhibition of NEAT1 and miR-324-5p partially reverses these effects and modulates KCTD20 expression. Overall, these findings highlight the NEAT1/miR-324-5p/KCTD20 axis as a novel regulatory pathway and a potential therapeutic target for human glioma [133].

In the realm of pediatric brain tumors, circRNAs emerge as novel regulators of tumorigenesis, modulating critical signaling pathways implicated in tumor initiation and growth. For example, upregulated circ-SKA3 and circ-DTL promote tumor cell proliferation and survival, migration capacity, and invasion ability in medulloblastoma cells, highlighting the diverse and context-dependent roles of ncRNAs in pediatric neuro-oncology [134].

### 3.9. Neurodevelopmental Psychiatric Disorders

**Autism Spectrum Disorder (ASD)** is a neurodevelopmental disorder characterized by persistent deficits in social communication and interaction, as well as restricted and repetitive patterns of behavior, interests, or activities. Symptoms typically emerge during early childhood and vary widely in severity, ranging from mild to severe impairment. ASD involves a complex interplay of genetic, synaptic, developmental, immune, epigenetic, and metabolic factors. Genetic variants affecting synaptic function and neuronal development are key contributors, alongside immune dysregulation, epigenetic modifications, and oxidative stress.

Partial loss of miR-137 in heterozygous knock-out mice leads to dysregulated synaptic plasticity, repetitive behavior, and impaired learning and social behavior. Elevated levels of the miR-137 target, Phosphodiesterase 10a (Pde10a), were found in these mice. Treatment with the PDE10A inhibitor papaverine or knockdown of Pde10a improved these deficits [135]. Mutations in the *SHANK* genes, which encode postsynaptic scaffolding proteins, are associated with a range of neurodevelopmental disorders, including ASD. Both *SHANK* genes and miR-137 are expressed at the synapse, influence neuronal development, and are strongly linked to neurodevelopment. This evidence suggests that *SHANK* genes might be targets of miR-137. Indeed, miR-137 directly targets the 3′UTR of *SHANK2* in a site-specific manner. Overexpression of miR-137 in mouse primary hippocampal neurons significantly reduces endogenous SHANK2 protein levels without affecting mRNA levels. Conversely, inhibiting miR-137 increases Shank2 protein expression, indicating that miR-137 regulates *SHANK2* expression by repressing protein translation rather than inducing mRNA degradation [136]. These findings indicate that miR-137 is crucial for postnatal neurodevelopment and suggest its dysregulation may contribute to human neuropsychiatric disorders.

NEAT1 and TUG1 lncRNAs are implicated in the pathogenesis of ASD through their roles in gene regulation. NEAT1, associated with paraspeckles in the nucleus, influences mRNA retention and has been linked to brain-derived neurotrophic factor (BDNF) expression. Elevated NEAT1 may contribute to ASD via the miR-497/BDNF pathway. TUG1 is a lncRNA with spatiotemporal differential expression in brain regions, identified in dispersed cells in the neocortex of adult brains. TUG1 regulates adjacent gene expression and is upregulated in ASD patients. TUG1 exerts inhibitory effects on miR-9, an evolutionary conserved miRNA whose overexpression has been associated with behavioral deficits in animals. Additionally, mir-9 also influences the proliferation, migration, and differentiation of neural progenitor cells [66,137].

Antisense lncRNA Shank2-AS is upregulated in patients with ASD, while its sense gene *SHANK2* is downregulated. SHANK2-AS can form a dsRNA with SHANK2 to inhibit its expression, impacting neuron structure and growth. Overexpression of SHANK2-AS reduces neurite numbers and lengths, inhibits neuron proliferation, and promotes apoptosis, contributing to ASD development. Studies on *SHANK2* mutant mice show ASD-like behaviors, highlighting the importance of SHANK2-AS in synaptic function and ASD pathology [138].

Similarly, the BDNF-AS, as a naturally occurring antisense RNA, regulates the expression of BDNF, which has essential roles in the pathoetiology of neurodevelopmental diseases, incl. ASD [139].

**Schizophrenia** is a chronic and severe psychiatric disorder characterized by disturbances in thought processes, perceptions, emotions, and behavior. Individuals with schizophrenia often experience hallucinations, delusions, disorganized thinking, and impairments in social and occupational functioning. Schizophrenia involves intricate molecular mechanisms, including disruptions in neurotransmitter systems like dopamine and glutamate, alterations in synaptic function, changes in neurodevelopmental processes, and genetic susceptibility. Dysfunction in various molecular pathways, such as neuroinflammation, oxidative stress, and mitochondrial dysfunction, also contribute to the pathogenesis of schizophrenia.

MiR-132 expression in the prefrontal cortex is developmentally regulated and linked to critical processes during adolescence. This microRNA, controlled by cyclic AMP-responsive element binding and NMDA, is significantly downregulated in individuals with schizophrenia. Gene expression analysis identified 26 upregulated miR-132 target genes, including *DNMT3A*, *GATA2*, and *DPYSL3*, which show altered expression in schizophrenia. Consistent with NMDA receptor hypofunction in schizophrenia, an NMDA antagonist given to mice downregulated miR-132 in the prefrontal cortex. Pharmacological inhibition of NMDA receptor signaling during a key postnatal period also downregulates miR-132 [140].

MiR-134 was found to be significantly decreased in peripheral blood mononuclear cells from schizophrenic patients and increased upon anti-psychotic treatment [141]

In contrast, the largest GWAS meta-analysis of schizophrenia to date, which systematically characterized key miRNAs associated with neurodevelopment and synaptic transmission, also identified elevated miR-137 in its target gene set [142].

A study investigated the expression of lncRNAs in the peripheral blood of schizophrenic patients compared to sex- and age-matched healthy controls using quantitative real-time PCR. It found that FAS-AS1, PVT1, and TUG1 were significantly downregulated in schizophrenic patients, while THRIL was upregulated. GAS5, NEAT1, and OIP5-AS1 showed no significant differences overall but were associated with schizophrenia in female subjects. The results suggest that lncRNAs may play a role in the pathogenesis of schizophrenia and could potentially serve as biomarkers for the disorder [143,144,145].

**Depression** is a mood disorder characterized by persistent feelings of sadness, hopelessness, and a lack of interest or pleasure in activities. It can affect how a person thinks, feels, and handles daily activities, often leading to a variety of emotional and physical problems. Depression can vary in severity, from mild to severe, and may be accompanied by symptoms such as changes in appetite, sleep disturbances, fatigue, and difficulty concentrating.

The molecular mechanisms of depression involve complex interactions among various neurotransmitters, hormones, and brain regions. Key players include neurotransmitters like serotonin, dopamine, and norepinephrine, as well as the hypothalamic–pituitary–adrenal (HPA) axis, which regulates stress response. Dysregulation of these systems can lead to alterations in neuroplasticity, inflammation, and synaptic function, contributing to the development and persistence of depressive symptoms.

Dysregulation of miRNAs and lncRNAs contributes to neurotrophic signaling deficits and synaptic remodeling in depression. MiR-16 has been associated with major depressive disorder (MDD) via regulation of the expression of the *serotonin transporter (SERT)* gene, and it is significantly lower in the CSF in patients compared to the healthy controls [146]. Elevated levels of BDNF-AS have been implicated in the pathophysiology of depression. BDNF-AS modulates BDNF expression, neurogenesis, and synaptic transmission in depression-related brain regions [147].

## 4. Diagnostic and Therapeutic Implications of ncRNAs

The dysregulation of ncRNAs in brain disorders offers potential diagnostic and therapeutic opportunities. NcRNAs can serve as biomarkers for disease diagnosis, prognosis, and treatment response, owing to their stability in bodily fluids such as blood, cerebrospinal fluid, and urine. Profiling of circulating miRNAs, lncRNAs, and circRNAs may facilitate the development of minimally invasive diagnostic tests for early detection and monitoring of brain disorders.

Furthermore, targeting dysregulated ncRNAs holds promise for the development of novel therapeutic interventions aimed at modulating disease progression and improving clinical outcomes, enabling personalized medicine approaches tailored to individual patients.

Strategies for ncRNA-based therapeutics include antisense oligonucleotides (ASOs), small interfering RNAs (siRNAs), and viral vectors for gene delivery.

Antisense oligonucleotides (ASOs) and small interfering RNA (siRNA) are two prominent classes of nucleic acid-based molecules utilized for gene regulation and therapeutic interventions. ASOs, typically single-stranded DNA or RNA molecules, exert their effects by binding to complementary mRNA sequences, thereby inhibiting translation, inducing mRNA degradation via RNase H-mediated cleavage, or modulating alternative splicing [148].

In contrast, siRNAs are double-stranded RNA molecules that guide the RNA-induced silencing complex (RISC) to target mRNAs with sequence complementarity. Once bound, the RISC cleaves the target mRNA, leading to its degradation and subsequent inhibition of protein expression [149]. ASOs are designed to target specific mRNA sequences based on sequence complementarity, while siRNAs target mRNAs through perfect or near-perfect complementarity within the coding region. Both ASOs and siRNAs can be delivered into cells using various methods, such as lipid nanoparticles or viral vectors, but ASOs often exhibit longer-lasting effects compared to siRNAs [150,151,152]. Despite their differences, both ASOs and siRNAs represent powerful tools for gene regulation and hold promise for therapeutic applications in various diseases.

Clinical trials have shown promising results for ASO-based therapies in neurodegenerative diseases such as spinal muscular atrophy (SMA) and ALS, highlighting their potential for treating neurological disorders [153].

Despite the growing interest in ncRNAs as diagnostic and therapeutic targets in neurological and psychiatric disorders, several challenges remain to be addressed. These include the development of robust methodologies for ncRNA detection and quantification, elucidation of the functional roles of specific ncRNAs in disease pathogenesis, and optimization of delivery systems for ncRNA-based therapeutics. Additionally, the complexity of ncRNA-mediated regulatory networks in the CNS poses challenges for deciphering their precise mechanisms of action and downstream effects on gene expression.

Future research directions in the field of ncRNAs and brain disorders should focus on integrating multi-omics approaches, including genomics, transcriptomics, proteomics, and metabolomics, to gain comprehensive insights into the molecular mechanisms underlying disease pathogenesis. Moreover, collaborative efforts between researchers, clinicians, and pharmaceutical companies are essential for translating basic science discoveries into clinically relevant diagnostic tools and therapeutic interventions for patients.

## 5. Challenges and Perspectives

NcRNAs play crucial roles in the regulation of gene expression and cellular processes in the brain, contributing to various physiological functions and pathological conditions. Their mechanisms of action in the brain are diverse, encompassing both transcriptional and post-transcriptional regulatory processes. NcRNAs are implicated in the pathogenesis of neurological and neuropsychiatric disorders, providing new avenues for understanding disease mechanisms and developing targeted therapies. This review focuses on the most studied ncRNAs related to brain disorders, for which mechanistic insights have been elucidated. While numerous studies have identified differentially expressed ncRNAs in various pathological conditions, the specific mechanisms of action and downstream signaling pathways of these ncRNAs remain largely unexplored. It is unclear whether the altered expression of these ncRNAs significantly impacts pathogenesis or merely reflects a consequence of the disease state; thus, determining whether these expression changes are causative or consequential is essential. Understanding the precise roles and regulatory mechanisms of ncRNAs is crucial for the development of therapeutic strategies, given that ncRNAs can have pleiotropic effects and off-target side effects must be avoided.

The clinical relevance of findings in ncRNA research is promising but still faces significant challenges, particularly concerning sample sizes and study reproducibility. While numerous studies have identified specific ncRNAs associated with disease processes, such as miR-34a in Alzheimer’s disease or BACE1-AS in amyloid-β production, many of these studies are based on small sample sizes. This limitation raises concerns about the statistical power and generalizability of the results. Small sample sizes increase the risk of type I and type II errors, potentially leading to false positives or overlooking crucial ncRNA functions. Moreover, the heterogeneous nature of clinical samples and the variability in experimental conditions further complicate the translation of these findings into clinical practice. For ncRNA research to achieve its potential in clinical applications, larger, well-designed studies with rigorous validation are imperative.

Future research should prioritize elucidating the pathways and interactions through which ncRNAs influence disease processes, as this could reveal novel therapeutic targets and enhance our understanding of brain pathologies. Despite the insights gained from miRNA profiling studies, it is essential to acknowledge the inherent limitations in the normalization of miRNA expressions, particularly in biofluids, due to challenges in identifying suitable housekeeping reference genes. Furthermore, while pathway analyses provide valuable insights into potential functional implications of dysregulated miRNAs, further investigations into their downstream coding RNA targets are warranted to elucidate their precise roles in disease pathophysiology.

Additionally, developing comprehensive databases of the various types of ncRNAs in the brain would be immensely beneficial for advancing research in this field. Such databases would serve as centralized repositories of information, cataloging the diverse ncRNAs identified in the brain along with their expression patterns, functions, and associations with specific brain regions and pathological conditions. These resources would provide researchers with valuable insights into the complex regulatory networks governed by ncRNAs. A well-curated database would include detailed annotations of ncRNAs, including their sequence information, biogenesis, molecular interactions, and regulatory mechanisms. It would also integrate data from various high-throughput techniques such as RNA sequencing, microarrays, and in situ hybridization, offering a comprehensive view of ncRNA expression profiles across different brain tissues and developmental stages. Furthermore, the database could link ncRNAs to their target genes and pathways, elucidating their roles in cellular processes and disease states.

Such a database would facilitate the identification of ncRNAs with potential diagnostic and therapeutic value, enabling researchers to prioritize candidates for further experimental validation. It could also support the development of computational tools for predicting ncRNA functions and interactions, advancing our understanding of their contributions to brain function and pathology. Moreover, an accessible and user-friendly database would promote data sharing and collaboration among researchers, accelerating the pace of discovery in the field of neurobiology.

## Figures and Tables

**Table 1:** MiRNAs implicated in molecular and cellular events in the brain, their targets, and their roles in pathological processes.

miRNA	Target	Cellular/Molecular Events	Disorders/Pathological Conditions
miR-132	*MeCP2* *p250GAP* *CREB* *FOXO3* *RhoA* *GABAR* *EPAC1* *Il-6* *p300* *FOXO3* *AchE*	Neurite outgrowth Dendritic arborization Synapse formation Synaptic plasticity Neuronal excitability Glia activation Apoptosis Oxidative stress Inflammation	Cognitive deficit Neurodegeneration Neurodevelopmental disorders Stroke Traumatic brain injury
miR-124	*Sox9* *PTBP1* *LIMK1* *NRXN1* *KCC2* *C/EBP-α* *GFAP* *CDK6* *BCL2L12* *MMP-9* *SNAI2*	Neural stem cell maintaining Neural differentiation Synaptic plasticity Neuronal connectivity Neuroinflammation Tumor suppression	Cognitive decline Neurodegeneration Stroke Brain tumors
miR-137	*Ezh2* *Sox2* *Tbr2* *NRG1* *GRIN2A* *PSD95*	Neurogenesis Synaptic function Plasticity	Cognitive dysfunction
miR-155	*SOCS1* *SHIP1* *Claudin 1* *ZO-1*	Neuroinflammation BBB integrity	Neurodegenerative disorders Cerebral ischemia
miR-9	*TLX* *FOXG1* *REST* *NFkβ* *GFAP* *HDAC5* *MECP2*	Neurogenesis Neural differentiation Neuroinflammation Chromatin remodeling Transcription activity	Neurodegeneration Stroke Traumatic brain injury
miR-134	*LIMK1* *CREB* *Pumilio* *DCX*	Synaptic plasticity Dendritic spine morphology GABA-ergic signaling	Epilepsy Schizophrenia
miR-34a	*NFkb* *STAT1* *c-Fos* *CREB* *P53*	Synaptic plasticity Glycolysis Oxidative phosphorylation	Neurodegeneration
miR-21	*PTEN* *PDCD4*	Oncogenic effect	Glioblastoma
miR-206	*HDAC4, BDNF, MEF2*	Translation, protein aggregation, oxidative stress Regeneration of neuromuscular synapses	ALS
miR-16-5p	*BDNF*	Apoptosis	Huntington’s disease

**Table 2:** LncRNAs implicated in molecular and cellular events in the brain, their targets, and their roles in pathological processes.

lncRNA	Target	Cellular/Molecular Events	Disorders/Pathological Conditions
BACE1-AS	*BACE1*	Aβ production	Alzheimer’s disease
NEAT1	Paraspeckels SFPQ	Transcriptional activity RNA processing mRNA splicing Cell cycle progression EMT DNA damage response pathways	Brain tumors Stroke
HOTAIR	Chromatin-modifying complexes—PRC2, LSD1	Histone methylation and acetylation Neuroinflammatory responses	Neurodevelopmental disorders Neurodegeneration Multiple sclerosis Stroke
SNHG1	Molecular sponge of miR-125b-5p, miR-216a-3p, miR-7, miR-194	Neuroinflammation Apoptosis Autophagy	Parkinson’s disease Glioma
MALAT1	Recruitment of SR family pre-mRNA-splicing factor	Regulation of gene expression, alternative splicing, epigenetic modifications Synapse formation Oxidative stress, apoptosis, neuroinflammation	Neurodegeneration AD, PD Brain tumors Stroke
GAS5	Inhibition of TRF4 by binding to PRC2	Inhibition of M2 polarization	Multiple sclerosis
BC1	Translational repressor in ImGluR-stimulated pathways	Synaptic transmission	Epilepsy
MEG3	miRNA sponge	Tumor suppressor Cell proliferation, apoptosis, and autophagy.	Glioma
TUG1	miR-9 inhibition	Proliferation, migration, differentiation of NPC	ASD Schizophrenia
BDNF-AS	Inhibits BDNF expression	Neuronal growth and differentiation, synaptic plasticity	Neurodevelopmental diseases
SHANK-AS	Inhibit *SHANK2* expression	Neurite number and length, apoptosis	Neurodevelopmental diseases
KCNQ1OT1	Binds PRC2 and G9a	Epigenetic regulation of immune cells functions and inflammatory responses	Multiple sclerosis
